# Inhibition of endosomal sequestration of basic anticancer drugs: influence on cytotoxicity and tissue penetration

**DOI:** 10.1038/sj.bjc.6603010

**Published:** 2006-02-21

**Authors:** C M Lee, I F Tannock

**Affiliations:** 1 Division of Applied Molecular Oncology and Department of Medical Oncology and Hematology, Princess Margaret Hospital/University of Toronto, 610 University Avenue, Toronto, Ontario, Canada M5G 2M9

**Keywords:** basic anticancer drugs, tissue penetration, chloroquine, omeprazole, endosomal pH

## Abstract

The basic drugs doxorubicin and mitoxantrone are known to be concentrated in acidic endosomes of cells. Here, we address the hypotheses that raising endosomal pH with the modifying agents chloroquine, omeprazole or bafilomycin A might decrease sequestration of anticancer drugs in endosomes, thereby increasing their cytotoxicity and availability for tissue penetration. Chloroquine, omeprazole and bafilomycin A showed concentration-dependent effects to raise endosomal pH, and to inhibit sequestration of doxorubicin in endosomes. Chloroquine and omeprazole but not bafilomycin A decreased the net uptake of doxorubicin into cells, but there was no significant effect on uptake of mitoxantrone. Omeprazole and bafilomycin A increased the cytotoxicity of the anticancer drugs for cultured cells, as measured in a clonogenic assay, whereas chloroquine had minimal effects on cytotoxicity despite reduced uptake of doxorubicin. Omeprazole but not chloroquine or bafilomycin A increased the penetration of anticancer drugs through multicellular layers of tumour tissue. We conclude that modifiers of endosomal pH might increase therapeutic effectiveness of basic drugs by increasing their toxicity and/or tissue penetration in solid tumours.

Most studies on causes of the limited success of chemotherapy for solid tumours have concentrated on cellular and molecular properties of tumour cells that lead to intrinsic or acquired drug resistance. While these mechanisms are important, solid tumours have a poorly formed vasculature and an additional explanation for the lack of effectiveness of many anticancer drugs may be their limited ability to penetrate through multiple cell layers of the extravascular space to reach all of the tumour cells (Durand, 1989; Jain, 1990; Hicks *et al*, 1997; Phillips *et al*, 1998; Lankelma *et al*, 1999; Tunggal *et al*, 1999; Tannock *et al*, 2002). The multicellular layer (MCL) model allows tumour cells to be grown in culture with many properties of tumours *in vivo*, including desmosomes between cells and an extracellular matrix (Hicks *et al*, 1997; Minchinton *et al*, 1997; Phillips *et al*, 1998; Tunggal *et al*, 1999, 2000; Cowan and Tannock, 2001; Tannock *et al*, 2002). Multicellular layers provide a simple, direct and quantitative means for measuring the penetration of drugs through solid tissue. The compound of interest is added to one side of the MCL and its appearance on the other side is measured by appropriate analytical methods. Using this method, we and others have demonstrated limited penetration of several anticancer drugs through solid tissue (Phillips *et al*, 1998; Tunggal *et al*, 1999; Tannock *et al*, 2002). Strategies that decrease the uptake of drugs into cells, such as high levels of folate in the presence of methotrexate or expression of P-glycoprotein in the presence of doxorubicin were found to increase tissue penetration, suggesting that tissue penetration is largely through the extracellular matrix (Tunggal *et al*, 2000; Cowan and Tannock, 2001).

Basic compounds such as doxorubicin and mitoxantrone are membrane permeable in their neutral form but relatively impermeable when protonated. Inside cells, basic drugs enter acidic cellular compartments, such as endosomes, where they become protonated and are sequestered (Mayer *et al*, 1986; Schindler *et al*, 1996; Hurwitz *et al*, 1997). In a series of papers, Simon and his co-workers (Schindler *et al*, 1996; Altan *et al*, 1998; Simon 1999; Rajagopal and Simon, 2003) have documented expression of multidrug-resistance transporters on endosomal membranes, and have shown increased sequestration of drugs in these compartments in drug-resistant cells. This sequestration decreases the availability of anticancer drugs for their cellular target (DNA in the nucleus). Depending on the rate of recycling of endosomes to fuse with the plasma membrane and release their contents extracellularly, it may also lead to less drug being available extracellularly for penetration to more distant cells.

The trapping of basic drugs in the acidic compartments of cells might be inhibited by decreasing the pH gradient across the intracellular endosomal membranes. Here, we describe two approaches to increasing endosomal pH by coadministration of other agents that are in clinical use: (i) a competing basic compound, chloroquine (used for treatment of malaria), which enters the acidic compartments and becomes protonated, thereby raising the pH in the endosomes (Poole and Ohkuma, 1981); (ii) omeprazole (used to reduce stomach acidity), which inhibits proton pumps including that in the endosomal membrane (Mattsson *et al*, 1991). We also include experiments with bafilomycin A, a more specific inhibitor of the endosomal proton pump (Mattsson *et al*, 1991), although this compound would be too toxic to use clinically.

In the present study, we address the following hypotheses: (i) that raising endosomal pH will decrease sequestration in endosomes of basic anticancer drugs such as doxorubicin and mitoxantrone; (ii) that decreased endosomal sequestration will increase cytotoxicity of these drugs and (iii) that decreasing endosomal sequestration will decrease cellular uptake of the anticancer drugs and hence allow them to better penetrate through tumour tissue.

## MATERIALS AND METHODS

### Cells and drugs

Experiments were performed using the mouse mammary sarcoma cell line, EMT-6 (obtained originally from Dr R Sutherland, University of Rochester, Rochester, NY, USA) and the human mammary carcinoma, MCF-7 obtained from American Type Culture Collection (Rockville, MD, USA). Cells were grown as monolayers in *α*-MEM (Sigma Chemical Co., St Louis, MO, USA) supplemented with 10% foetal bovine serum (Cansera, Toronto, Ontario, Canada) at 37°C in a humidified atmosphere of 95% air plus 5% CO_2_. Experiments on dispersed cells were performed using exponentially growing cells. Tests were performed routinely to ensure that cells were free of mycoplasma.

Doxorubicin and mitoxantrone were obtained from the hospital pharmacy. Chloroquine was purchased from Sigma (St Louis, MO, USA) and dissolved in phosphate-buffered saline. Omeprazole was obtained in powder form from AstraZeneca (Molndal, Sweden) and dissolved in ethanol. Bafilomycin A was obtained from Sigma (St Louis, MO, USA) and dissolved in DMSO.

### Measurement of endosomal pH

The influence of chloroquine, omeprazole and bafilomycin A on endosomal pH was measured as follows. Cells (10^6^ ml^−1^) were treated with varying concentrations of these agents, or with the vehicle solution. They were incubated for 3 h with dextran-fluorescein-tetramethylrhodamine 10 000 MW, anionic (FITC/TMR-dextran, Molecular Probes, Inc., Eugene, OR, USA), which is taken up into endosomes, followed by exposure to media for 2 h. Fluorescence was measured using a Coulter Epics Elite flow cytometer (Beckman Coulter, Miami, FL, USA) equipped with an argon laser emitting at 488 nm. The argon laser was used to excite FITC and TMR with emission evaluated at 525 nm (pH-dependent) and 575 nm (pH-independent), respectively. Calibration of fluorescence measurements was performed using the ionophore nigericin (Sigma, St Louis, MO, USA) in buffers of known pH (Poole and Ohkuma, 1981). This allows equilibration of all the internal compartments of cells to the pH of the incubating buffer. A curve was generated that demonstrated the relationship between FITC fluorescence emission ratio and pH.

### Fluorescence microscopy

The distribution of doxorubicin in cells, in the presence or absence of agents that might influence endosomal pH, was evaluated by fluorescence microscopy. Cells attached to a chambered cover-glass were pretreated with chloroquine, omeprazole or bafilomycin A and then incubated in media containing 3.5 *μ*M doxorubicin for 2 h. At the end of the incubation, the drugs were washed out and the cover-glass was placed on the microscope stage and fluorescence signal was recorded using a Zeiss Axiovert 200 M fluorescence inverted microscope, equipped with a 530–560 nm excitation and a 573–647 nm emission filter set. The presence of fluorescent doxorubicin in cellular compartments was captured with a Roper Scientific CoolSnap HQ CCD camera, and false-coloured red.

To visualise endosomes, the cells were also exposed to the pH-sensitive endosomal dye, LysoSensor Yellow/Blue DND-160 (Molecular Probes, Inc., Eugene, OR, USA) at a concentration of 5 *μ*M for 15 min (Hurwitz *et al*, 1997). The fluorescent signal was measured with excitation at 360 nm and emission at 420 nm and false-coloured blue. Evidence for colocalisation of doxorubicin and lysoSensor was sought by overlaying images of the same cells, and seeking fusion of the colours of the two images (purple).

### Drug uptake into cells

The uptake into cells of radiolabelled ^14^C-doxorubicin (Amersham, England) and ^3^H-mitoxantrone (Moravek Chemicals, Brea, CA, USA) was studied using a spin-through-oil technique (Keyes *et al*, 1987; Cowan *et al*, 1992). Stirred suspensions of single cells were pretreated in the presence or absence of chloroquine or omeprazole (100 *μ*M and 1 mM) or 100 nM bafilomycin A for 1 h; radiolabelled doxorubicin (0.27 *μ*M; 0.15 *μ*Ci 10 ml^−1^) or mitoxantrone (67 nM; 2 *μ*Ci 10 ml^−1^) was then added to the suspension. Aliquots (100 *μ*l) were removed as a function of time (up to 5 h) and layered on top of a mixture of dibutylphthalate and corn oil (4 : 1) in microcentrifuge tubes. The tubes were then spun at 14 000 r.p.m. for 5 min, and the cells were pelleted at the bottom of the tube. The medium and then the oil were aspirated and the cell-associated radioactivity was determined by liquid scintillation counting.

### Assessment of cytotoxicity

Cytotoxicity was evaluated by a colony-forming assay. Single cell suspensions were treated in glass polyshell vials with 1.8 *μ*M doxorubicin or 1 *μ*M mitoxantrone in the presence or absence of 100 *μ*M chloroquine, 100 *μ*M omeprazole or 100 nM bafilomycin A. The vials were placed in a water bath at 37°C, magnetically stirred and gassed with 95% air plus 5% CO_2_. Samples were removed as a function of time up to 5 h later, centrifuged, washed and plated in serial dilution in plastic tissue culture dishes containing 5 ml medium. Colonies generated 8–14 days later were stained with methylene blue and counted.

### Penetration of drugs through MCL

Multicellular layers (MCL)were generated by seeding exponentially growing cells on collagen-coated microporous Teflon membranes attached to culture plate inserts (Millicell-CM inserts, 3 *μ*m pore size, Millipore, Bedford, MA, USA). Prior to use in experiments, the MCL were examined by a light microscope to ensure uniform thickness. One or two randomly selected MCL per experiment were dissociated using trypsin to determine the total number of cells; MCL containing ∼3–4 × 10^6^ cells were used in the experiments. Solutions containing ^14^C-doxorubicin or ^3^H-mitoxantrone in 1% agar solution were added to one side of the MCL (compartment 1) and the membrane was then floated on a larger volume of stirred culture media (compartment 2). ^3^H- or ^14^C-sucrose was added and the penetration of sucrose was used as an internal control. Agar prevents convective motion from influencing penetration properties but does not inhibit drug transport. The concentration of the drug in compartment 2 as a function of time (up to 6 h) was measured by scintillation counting, and was expressed as a ratio (‘relative penetration’) of that achieved after the same time interval by penetration through the Teflon membrane in the absence of an MCL.

### Statistical analysis

Student's *t*-test was used for the determination of statistical significance when comparing outcome of different experimental conditions.

## RESULTS

### Endosomal pH

**Figure 1:**
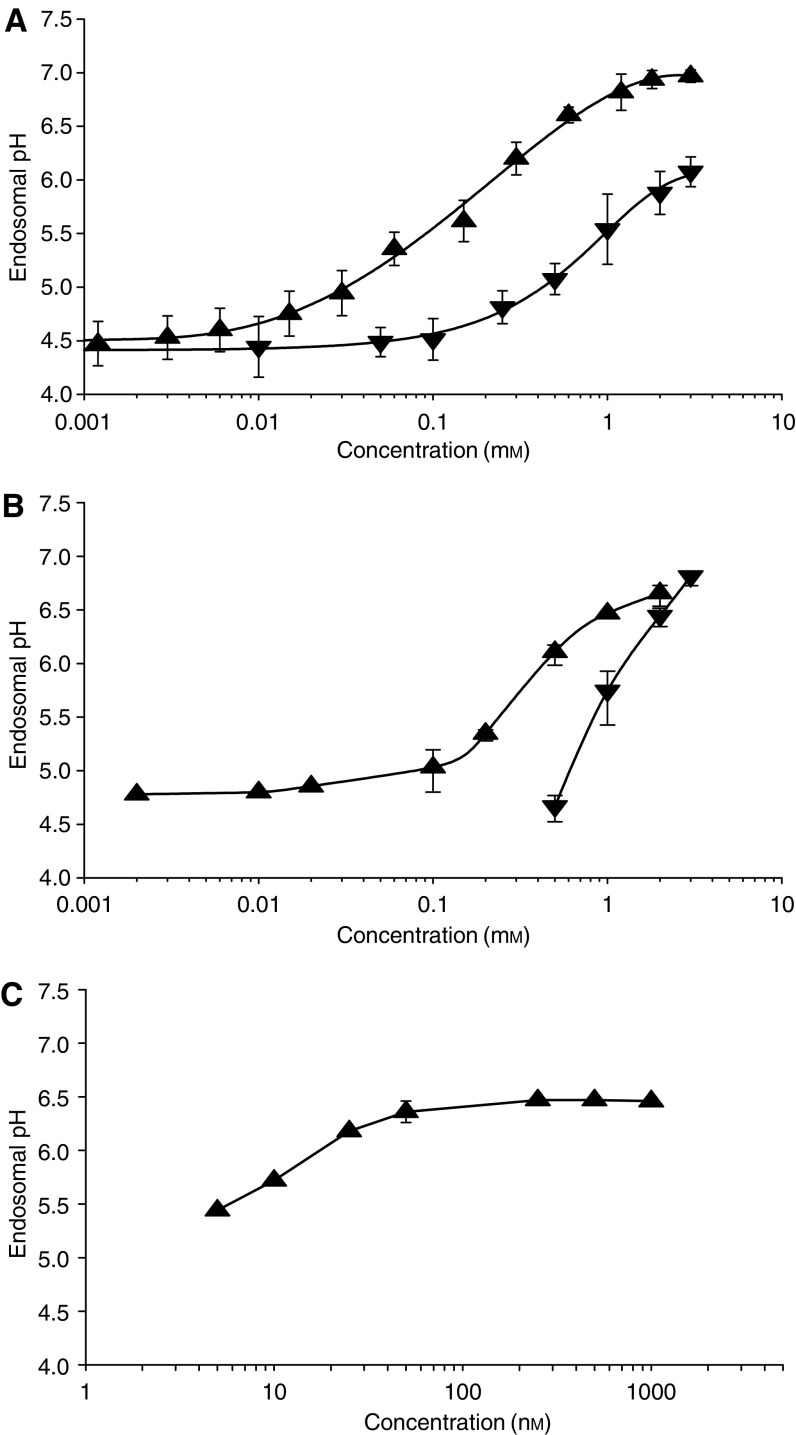
Endosomal pH measurements in (**A**) EMT-6 and (**B**) MCF-7 cells exposed to chloroquine (▴) or omeprazole (▾). Also indicated are the effects on endosomal pH of EMT-6 cells for the endosomal proton pump inhibitor bafilomycin A (**C**). Means and s.e.m. are shown for at least three independent measurements. (Where bars are not shown, s.e.m. is less than the height of the points.)

Chloroquine, omeprazole and bafilomycin A increased the endosomal pH in a dose-dependent manner by a maximum of approximately two pH units in both EMT-6 (Figure 1A) and MCF-7 cells (Figure 1B). Chloroquine was effective in changing the endosomal pH at a lower concentration than omeprazole. Figure 1C shows that bafilomycin A was effective in raising endosomal pH at much lower concentration with maximal effects observed at a concentration of about 100 nM.

### Cellular uptake and localisation of anticancer drugs

**Figure 2:**
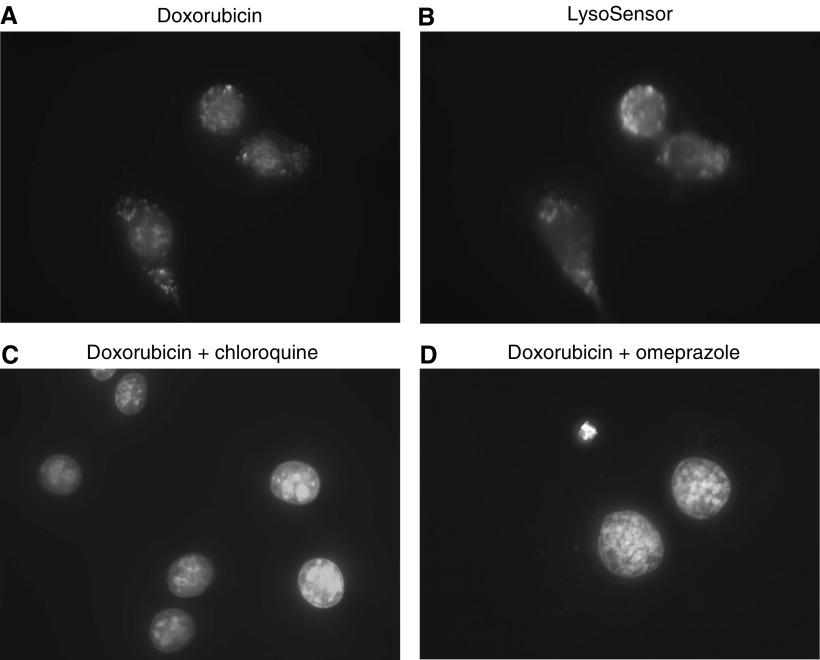
Fluorescent micrographs show EMT-6 cells treated with (**A**) doxorubicin (3.5 *μ*M), (**B**) the fluorescent pH indicator LysoSensor Yellow/Blue DND-160 which accumulates in acidic organelles, (**C**) doxorubicin and chloroquine 100 *μ*M and (**D**) doxorubicin and omeprazole 1 mM. Note decrease in fluorescence in endosomes due to doxorubicin in (C and D). Similar results were obtained for MCF7 cells. Direct comparison of doxorubicin uptake is not possible because of photobleaching.

Fluorescence micrographs show that doxorubicin was sequestered in a punctate pattern in the cytoplasm of both EMT-6 (Figure 2A) and MCF-7 cells (not shown) as well as being taken up into the nucleus, where it is known to target DNA. LysoSensor Yellow/Blue DND-160 is known to accumulate in acidic organelles such as endosomes (Figure 2B), and overlay experiments confirmed that doxorubicin colocalised with this fluorescent pH indicator (not shown). Chloroquine and omeprazole led to a reduction in doxorubicin fluorescence in endosomes (Figures 2C and D). Higher concentration (1 mM) of omeprazole than chloroquine was required to modify the cellular localisation of doxorubicin, consistent with the dose–response relationships for these agents to influence endosomal pH. Bafilomycin A at a concentration of 100 nM reduced doxorubicin fluorescence in endosomes to undetectable levels (not shown). Quantifying the amount of fluorescent doxorubicin in cells was not possible due to its photobleaching characteristics.

**Figure 3:**
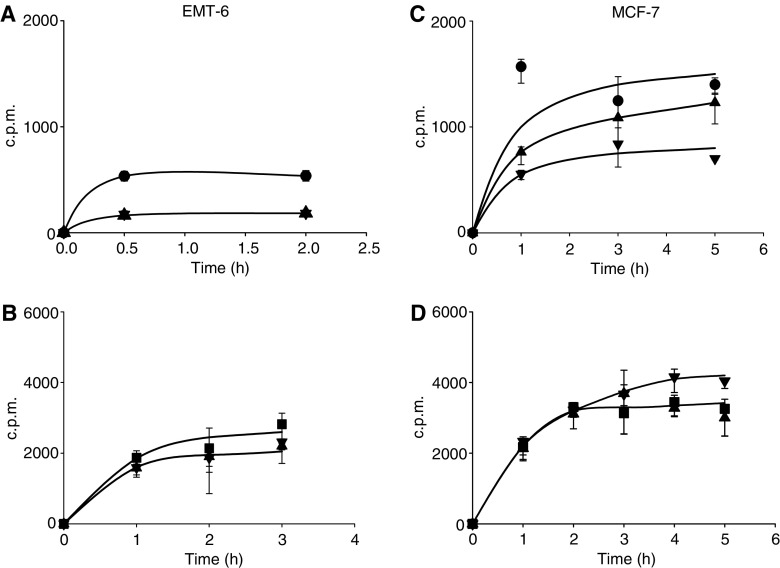
Time-dependent uptake of radiolabelled doxorubicin (**A** and **C**) and mitoxantrone (**B** and **D**) into EMT-6 cells (**A** and **B**) and MCF-7 cells (**C** and **D**). Doxorubicin alone (•), mitoxantrone alone (▪), drug plus chloroquine 100 *μ*M (▴) or omeprazole 100 *μ*M (▾). Mean and s.e.m. are shown for three independent experiments. Significant effects (*P*<0.001, Student's *t*-test) are seen for both modifiers in Figure 3A. (c.p.m.=counts per minute in cell pellet).

There were inconsistent effects of chloroquine or omeprazole to influence the total cellular uptake of the radiolabelled anticancer drugs (Figure 3). The total cellular accumulation of doxorubicin in EMT-6 cells was reduced by coadministration of chloroquine and by omeprazole at 100 *μ*M (Figure 3A), but (surprisingly) not at 1 mM (data not shown). Both modifiers reduced uptake of doxorubicin into MCF-7 cells, but there was no difference in the magnitude of the effect for concentrations of 100 *μ*M (Figure 3B) and 1 mM (not shown). There was no consistent effect for omeprazole or chloroquine (at either concentration) to influence the net uptake of mitoxantrone (Figure 3B and D). Bafilomycin A (100 nM) did not influence the uptake of doxorubicin into EMT-6 cells.

### Cytotoxicity of anticancer drugs

**Figure 4:**
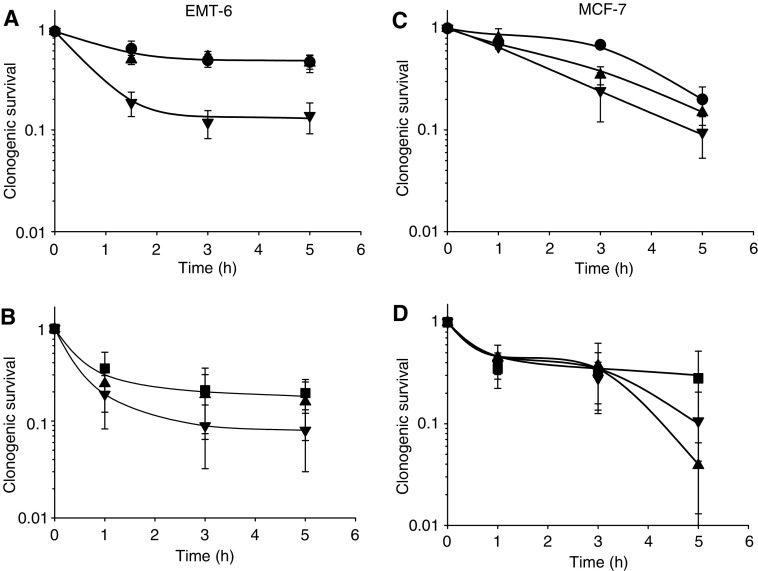
The effect of omeprazole and chloroquine on the toxicity of doxorubicin (**A** and **C**) and mitoxantrone (**B** and **D**) for EMT-6 (**A** and **B**) and MCF-7 (**C** and **D**) cells as determined by a clonogenic assay. Doxorubicin alone (•), mitoxantrone alone (▪), drug plus chloroquine 100 *μ*M (▴) or omeprazole 100 *μ*M (▾). Mean and s.e.m. are shown for three independent experiments. Chloroquine and omeprazole showed no cytotoxicity when used alone.

Exposure of cells to chloroquine (100 *μ*M), omeprazole (100 *μ*M or 1 mM) or bafilomycin A (100 nM) for up to 5 h was not toxic to cells, as determined in a colony-forming assay. Omeprazole (100 *μ*M) increased the cytotoxicity of doxorubicin for EMT-6 and MCF-7 cells, and of mitoxantrone for EMT-6 cells (Figure 4A–C), and had marked effects to increase toxicity of doxorubicin when used at a concentration of 1 mM (data not shown). Chloroquine had minimal effects on the cytotoxicity of doxorubicin and mitoxantrone for both EMT-6 and MCF-7 cells (Figure 4). Bafilomycin A (100 nM) increased the cytotoxicity of doxorubicin for EMT-6 cells (data not shown).

### Tissue penetration of anticancer drugs

Previous experiments have shown that both murine EMT-6 and human MCF-7 breast cancer cells produce MCL of consistent thickness (∼150 *μ*M). The penetration of radiolabelled mitoxantrone and doxorubicin through the Teflon membrane that is used to support the growth of MCL leads to 30–40% of equilibrium concentration in the receiving compartment at 4–6 h (Tunggal *et al*, 1999).

**Figure 5:**
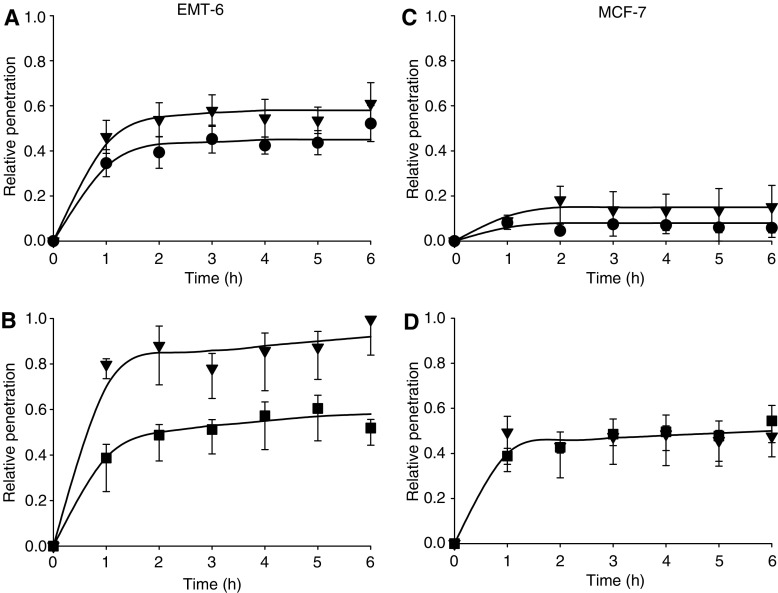
Penetration of radiolabelled doxorubicin (**A** and **C**) and mitoxantrone (**B** and **D**) through MCL derived from EMT-6 (**A** and **B**) and MCF-7 (**C** and **D**) cells relative to that through the Teflon membrane alone at each time interval. MCL were exposed to doxorubicin alone (•), mitoxantrone alone (▪) or drug plus omeprazole 100 *μ*M (▾). Mean and s.e.m. are shown for three independent experiments.

Tissue penetration relative to that through the cell-free membrane is shown in Figure 5. Omeprazole (100 *μ*M) increased the penetration of doxorubicin and mitoxantrone through MCL grown from EMT-6 cells (Figure 5A and B) (*P*<0.05 for Figure 5B). Smaller or no changes were observed with the penetration of doxorubicin and or mitoxantrone through MCL grown from MCF-7 cells (Figure 5C and D). Chloroquine (100 *μ*M) had little or no effect on penetration of doxorubicin for either cell lines, but improved the penetration of mitoxantrone through MCL derived from EMT-6 cells but not those from MCF-7 cells (data not shown). Bafilomycin A (100 nM) did not influence tissue penetration.

## DISCUSSION

In designing the present experiments, we established three related hypotheses: (i) that raising endosomal pH will decrease sequestration in endosomes of basic anticancer drugs such as doxorubicin and mitoxantrone; (ii) that decreased endosomal sequestration will increase cytotoxicity of these drugs and (iii) that decreasing endosomal sequestration will decrease net cellular uptake of the anticancer drugs and hence allow them to better penetrate through tumour tissue. Our results provide strong support for the first hypothesis and partial support for the second and third.

We elected to study chloroquine and omeprazole because they are in clinical use (for treatment of malaria and ulcer disease, respectively) and because quite high doses are tolerated *in vivo* (Lambers *et al*, 1984; Onyeji and Ogunbona, 2001). This would allow the rapid transfer of encouraging laboratory data to evaluation in a clinical trial. Bafilomycin A is not an agent that could be used clinically, but was included as a specific inhibitor of the endosomal proton pump.

The two cell lines used in these experiments express at most low levels of P-glycoprotein (data not shown) and are moderately sensitive to doxorubicin and mitoxantrone (Figure 4). However, contrary to the findings of Altan *et al* (1998), these cells contained acidified endosomes (Figure 1) and the basic drug doxorubicin localized within them (Figure 2). Each of the modifying agents increased endosomal pH in a dose-dependent manner in the two malignant cell lines that were studied (Figure 1). As expected, this was achieved at a lower concentration of bafilomycin A (∼100 nM), while concentrations of 100 *μ*M chloroquine or 1 mM omeprazole were needed to achieve the same effect. We expected less trapping of basic drugs in endosomes with raised pH and confirmed that there was direct correlation between the ability of modifying agents to raise endosomal pH and to reduce doxorubicin fluorescence in endosomes.

When basic anticancer drugs enter cells, there is likely to be competition for uptake into endosomes and into the nucleus. We have confirmed colocalisation of fluorescent doxorubicin with a fluorescent lysosomal probe, although the method does not allow quantitative comparison of drug concentration among cellular compartments because of the effect of different physical properties (e.g. pH) and binding (e.g. to DNA). We hypothesised that if uptake into endosomes was inhibited, either more drug would be available to the nucleus, leading to greater toxicity for a given level of cellular uptake, or (if equilibrium was achieved) there would be a decrease in net cellular uptake without change in the amount of drug reaching the cell nucleus or in cytotoxicity. We found that the modifiers caused relatively small changes in net cellular uptake of the anticancer drugs, so that concentrations of omeprazole and chloroquine that inhibit uptake of anticancer drugs into endosomes probably cause redistribution of doxorubicin and mitoxantrone within the cell. Chloroquine is also known to be an inhibitor of DNA repair, and there is recent evidence that it can upregulate p53-mediated apoptosis (Mohamed, 2005). We found that chloroquine had minimal effects on the cytotoxicity of the anticancer drugs (despite reduced net cellular uptake of doxorubicin), whereas concentrations of omeprazole or bafilomycin A that showed no cytotoxicity when used alone increased the cytotoxicity of the anticancer drugs (Figure 4). Whether the effect of omeprazole to increase cytotoxicity might be useful clinically would depend on relative effects against tumour and dose-limiting normal tissues.

Our third hypothesis, that the modifiers might improve the poor penetration of doxorubicin and mitoxantrone through tumour tissue was based on previous studies indicating that drug penetration was largely through the extracellular space and that mechanisms that decreased cellular uptake of drugs improved their tissue penetration (Tunggal *et al*, 2000; Cowan and Tannock, 2001). Decreased uptake of basic drugs into endosomes might be expected to lead to a decrease in the their total cellular accumulation, although endosomes are also known to fuse with the cell membrane and deposit their contents extracellularly (Schindler *et al*, 1996; Simon, 1999), so this will depend on the rate of endosomal recycling. The net uptake of doxorubicin, but not mitoxantrone was reduced by coadministration of either chloroquine or omeprazole (Figure 3), although effects of omeprazole were observed at doses below those found to have an effect on endosomal pH, which might be due to other mechanisms. There was no effect of bafilomycin A on cellular uptake of the anticancer drugs. Chloroquine is not only a weak base but also a known (if relatively poor) inhibitor of P-glycoprotein (Beck *et al*, 1988; Riffkin *et al*, 1996); hence, part of its effect to decrease cellular uptake of doxorubicin might be due to increased export across the cellular membrane.

We would expect improved penetration of anticancer drugs under conditions when net cellular uptake is inhibited, and our measured effects on cellular uptake were inconsistent and smaller than we had originally anticipated. We did find effects to improve tissue penetration using the MCL model, although these results were cell line-dependent and correlated imperfectly with the influence of the modifiers on net cellular uptake of the anticancer drugs. It is evident that multiple factors influence tissue penetration, including not only drug uptake but also unknown effects of modifying agents on the extracellular environment. Nonetheless, the demonstration of an ability to improve tissue penetration using strategies that enhance or maintain cytotoxicity to proximal cells has potential application to improve therapeutic index, since limited tissue penetration is unlikely to occur in well-vascularised dose-limiting normal tissues such as the bone marrow. The application of these results to improving tissue penetration *in vivo* will depend on additional factors, including the tissue penetration of the modifiers themselves. We have established recently a method of quantifying tissue gradients of doxorubicin from blood vessels in solid tumours (Primeau *et al*, 2005), and will investigate the use of modifying agents studied here to change those distributions in tumours. The effects on therapeutic index will depend also on the relative importance of quiescence *vs* limited drug distribution in leading to resistance of distal cells, and on possible effects of modifiers to redistribute anticancer drugs in cellular compartments of normal cells, thereby increasing toxicity.
